# Paediatric asthma and non‐allergic comorbidities: A review of current risk and proposed mechanisms

**DOI:** 10.1111/cea.14207

**Published:** 2022-07-28

**Authors:** Bronwyn K. Brew, Emma Caffrey Osvald, Tong Gong, Anna M. Hedman, Kirsten Holmberg, Henrik Larsson, Jonas F. Ludvigsson, Mwenya Mubanga, Awad I. Smew, Catarina Almqvist

**Affiliations:** ^1^ Department of Medical Epidemiology and Biostatistics Karolinska Institutet Solna Sweden; ^2^ National Perinatal Epidemiology and Statistics Unit, Centre for Big Data Research in Health and School of Clinical Medicine University of New South Wales Kensington New South Wales Australia; ^3^ Pediatric Allergy and Pulmonology Unit, Astrid Lindgren Children's Hospital Karolinska University Hospital Stockholm Sweden; ^4^ Child Health and Parenting (CHAP), Department of Public Health and Caring Sciences Uppsala University Uppsala Sweden; ^5^ School of Medical Sciences Örebro University Örebro Sweden; ^6^ Department of Pediatrics Orebro University Hospital Orebro Sweden

**Keywords:** anxiety, asthma, children, comorbidity, depression, obesity, sleep

## Abstract

It is increasingly recognized that children with asthma are at a higher risk of other non‐allergic concurrent diseases than the non‐asthma population. A plethora of recent research has reported on these comorbidities and progress has been made in understanding the mechanisms for comorbidity. The goal of this review was to assess the most recent evidence (2016–2021) on the extent of common comorbidities (obesity, depression and anxiety, neurodevelopmental disorders, sleep disorders and autoimmune diseases) and the latest mechanistic research, highlighting knowledge gaps requiring further investigation. We found that the majority of recent studies from around the world demonstrate that children with asthma are at an increased risk of having at least one of the studied comorbidities. A range of potential mechanisms were identified including common early life risk factors, common genetic factors, causal relationships, asthma medication and embryologic origins. Studies varied in their selection of population, asthma definition and outcome definitions. Next, steps in future studies should include using objective measures of asthma, such as lung function and immunological data, as well as investigating asthma phenotypes and endotypes. Larger complex genetic analyses are needed, including genome‐wide association studies, gene expression–functional as well as pathway analyses or Mendelian randomization techniques; and identification of gene–environment interactions, such as epi‐genetic studies or twin analyses, including omics and early life exposure data. Importantly, research should have relevance to clinical and public health translation including clinical practice, asthma management guidelines and intervention studies aimed at reducing comorbidities.

## Graphical abstract

We conducted a review of recent research regarding the comorbidity of asthma and non‐allergic diseases in children. There is increasing evidence for a number of co‐morbidites with a range of possible mechanisms including common genetic factors, early life risk factors and causal associations.

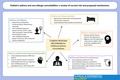

**Key Messages**

- Children with asthma are more likely to experience other non‐allergic diseases compared to children without asthma
- Proposed mechanisms for comorbidity include: causal associations, early life risk factors and common genetic factors
- More research is needed using rich, well‐defined datasets to elucidate possible points of prevention.

## 1 INTRODUCTION

Asthma comorbidities place an extra burden on healthcare systems,^1^ and the excess costs due to comorbidities are up to five times higher than asthma alone.^2^ Not only do asthma comorbidities increase financial and healthcare burden but they can also exacerbate asthma symptoms and lead to worse health outcomes.^3^ Understanding comorbidities in children is therefore important, especially with the current obesity epidemic potentially adding a further set of potential comorbidities such as Type 2 diabetes and sleep disordered breathing.^4^

Over a decade ago, a special paediatric asthma series in the European Respiratory Journal included a review entitled “Comorbidities of asthma during childhood: possibly important, yet poorly studied.”^4^ The authors concluded that knowledge of asthma comorbidities in childhood was lacking and that studies were urgently needed to address the prevalence and effects of comorbidities on asthma control and treatment.^4^ Since this seminal paper, a large and diverse number of studies have been published, improving knowledge about prevalence, risk and the impact of comorbidities. Of note, in 2016, the US National Health Interview Survey found that the prevalence of almost every 1 of the 41 diseases measured in that survey was higher in children with asthma compared to children without asthma including non‐allergic diseases such as mental health conditions (e.g. depression and prevalence difference [PD] 3.4%, 95% CI 1.8, 5.1), neurological conditions (e.g. attention deficit hyperactivity disorder, PD 4.5%, 95% CI 2.2, 6.7) and sleep issues (e.g. insomnia, PD 6.6%, 95% CI 4.4, 8.9).^5^

The goal of this review is to provide a critique of current evidence about non‐allergic comorbidities in paediatric asthma. In particular, to assess the extent of comorbidities and provide an understanding on the latest mechanistic research explaining why children with asthma are more at risk of non‐allergic comorbidities, and highlight knowledge gaps requiring further research. We did not include allergic comorbidities as these have been well studied for many years and the mechanisms have now been shown to be largely genetic in nature.^6^

## 2 METHODS

**FIGURE 1:**
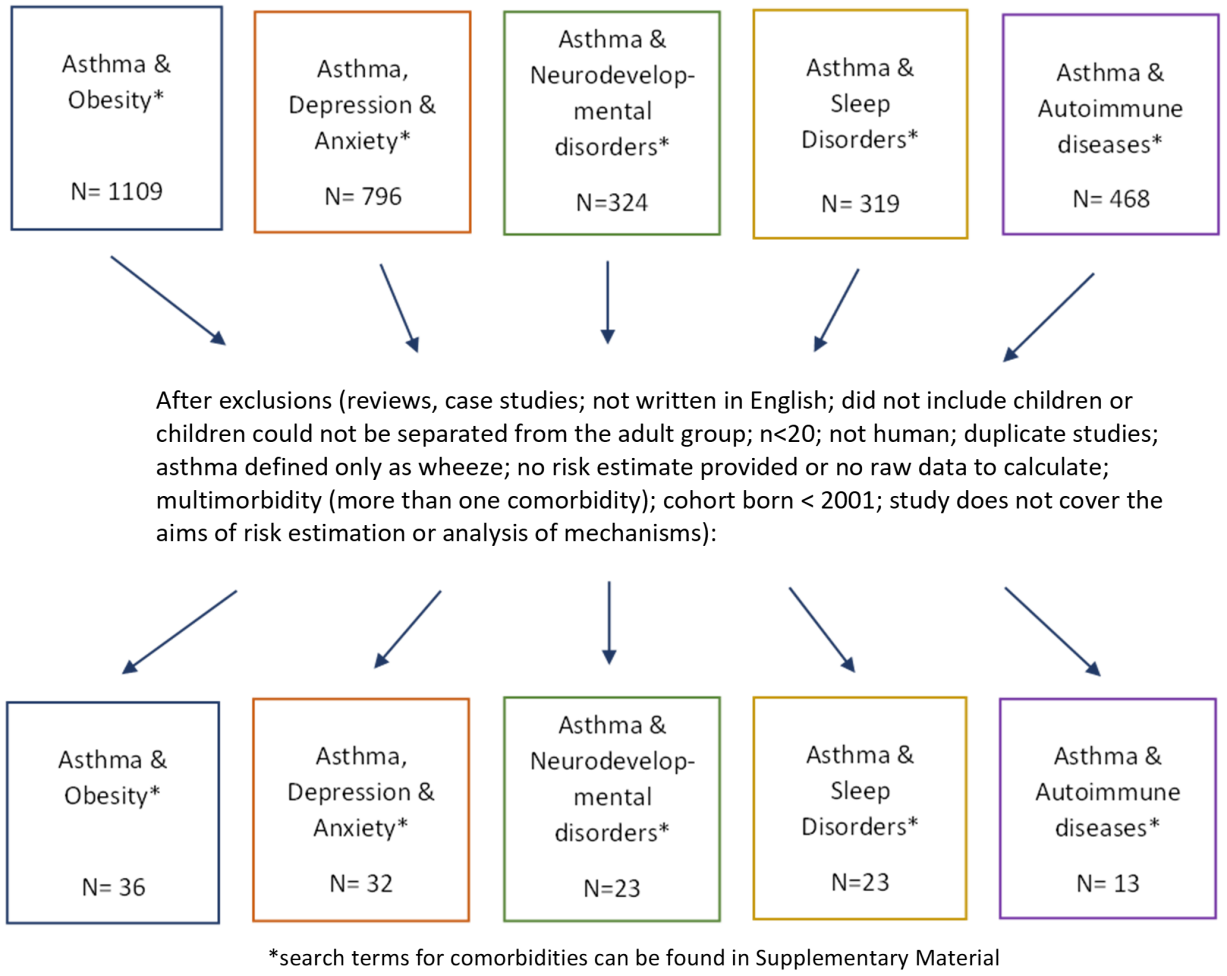
Results of MEDLINE and ePUB search 2016–2021, child and adolescent asthma and comorbidities: risk, symptom and mechanism studies.

Comorbidities were chosen as the most common non‐atopic and non‐communicable diseases reported in relation to asthma in children. We searched MEDLINE® and Epub Ahead of Print on 9 November 2021 for all articles in the last 5 years (2016–2021) conducted on children or adolescents that included ‘asthma’ and the comorbidity of interest in the abstract, title or author keywords (the exact search terms used for each comorbidity are found Table S1). Eight of the authors then reviewed the titles, abstracts and articles and excluded irrelevant articles, articles not in English or abstracts without full‐length articles. Figure 1 shows the results of the search and the exclusion criteria. Data were extracted according to an extraction template, and articles were checked by at least two authors. The focus of the data extraction included: (a) asthma as a risk factor for comorbidities (rather than comorbidities as a risk factor for asthma) expressed as an odds ratio, risk ratio, hazards ratio, linear β‐value and prevalence difference, or if these were not available, a p‐value signifying no difference in risk between children with asthma and those without; (b) symptom manifestations of asthma and comorbidities above and beyond each disease alone; and (c) mechanistic research on asthma and comorbidities.

## 3 RESULTS

### 3.1 Asthma and Obesity

Overweight and obesity are defined as the abnormal or excessive accumulation of fat that may impair health.^7^ According to a recent global study targeting 5‐ to 19‐year‐olds, the mean body mass index (BMI) and prevalence of obesity have increased globally, negatively affecting health and quality of life.^8^ Obesity is commonly associated with the onset of asthma, poor asthma control and the development of other comorbid conditions such as gastroesophageal reflux,^9^, ^10^ obstructive sleep apnea,^7^, ^9^ type 2 diabetes and an increased risk of recurrent hospitalization.^10^ Although in adult studies asthma severity is associated with obesity,^11^, ^12^ this association was not observed in a recent study in children.^13^ Studies suggest that the most common asthma phenotype associated with obesity is non‐allergic asthma with non‐type‐2 (Th2) inflammation.^14^, ^15^ Other phenotypes such as exercise‐induced asthma and non‐allergic asthma have not shown a consistent association with BMI in children.^16^, ^17^, ^18^

**FIGURE 2:**
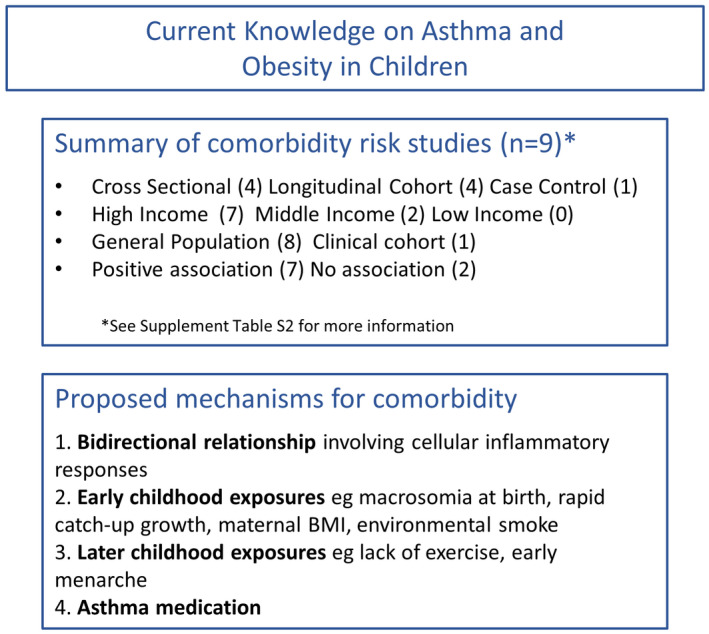
Current knowledge on asthma and obesity in children. Summary of study factors measuring obesity as a risk of asthma, and a summary of currently proposed mechanisms explaining the comorbidity of asthma and obesity.

Our search of publications revealed nine studies measuring the magnitude of the risk of obesity in children with asthma (Table S2). The majority of studies found a positive association between childhood asthma and obesity (*n* = 7), including three prospective studies showing that non‐obese children diagnosed with asthma are at a higher risk of developing obesity than those without asthma.^19^, ^20^, ^21^ A pooled cohort study of European countries (*n* = 21,130) reported similar findings of asthma and obesity comorbidity (HR 1.66, 95% CI, 1.18, 2.33) compared to large cross‐sectional studies in China and the USA (OR 1.51, 95% CI, 1.03, 2.21 and RR 1.67 [95% CI, 1.32, 2.11] respectively).^21^, ^22^, ^23^ These results are further supported by a recent meta‐analysis which found the pooled RR to be 1.47 (95% CI, 1.25, 1.72).^24^ Furthermore, comorbidity was higher in boys,^20^, ^21^ black children^25^ and physically inactive individuals (Figure 2).^22^, ^26^

The mechanisms linking asthma with obesity are unclear, but it is hypothesized that the two conditions may share common etiological pathways. Asthma is associated with increased systemic oxidative stress^27^ which is characterized by an imbalance between cellular antioxidant defences and overproduction of free radicals including increased reactive oxygen species.^28^ These free radicals cause oxidative injury to the lung and pro‐inflammatory cytokine release, thus promoting systemic inflammation.^29^ They are also involved in the control of body weight by exerting different effects on hypothalamic neurons, which control satiety and hunger behaviour.^30^ Children diagnosed with asthma tend to have higher age‐specific serum triglyceride levels and higher rates of insulin resistance than non‐asthmatics.^31^

Possible common early life exposure candidates, which are risk factors for both asthma and obesity, include maternal BMI,^32^, ^33^ early antibiotic use,^34^ mode of delivery,^34^ early feeding practices,^35^ poverty and environmental smoke.^36^, ^37^ High birth weight in early childhood is associated with both asthma and obesity suggesting a role of early BMI on later outcomes.^38^ Additionally, catch‐up growth, a period of faster weight gain during the first 2 years of life, has been associated with the risk of obesity^39^ and asthma separately.^35^, ^39^

Possible common candidates for comorbidity later in childhood include high BMI, lack of physical exercise and hormonal changes. Children with high BMI tend to be less physically active and are at risk of developing atopic sensitization and asthma in addition to obesity.^38^, ^40^ It has also been observed that asthma, especially when undiagnosed, poorly controlled or perceived by parents as a health risk may limit engagement in physical activity, thus increasing the risk of obesity.^41^, ^42^, ^43^ Early menarche increases the risk of obesity in adolescent girls with asthma.^40^, ^44^, ^45^ and has been shown to be associated with weight gain, lower lung function and increased severity of asthma symptoms.^44^

Medications used in the treatment of asthma may play an important role in the comorbidity of asthma and obesity in children, although research is conflicting. A US study found that the use of rescue medication such as short‐acting beta‐agonists reduced the risk of obesity independent of asthma diagnosis.^20^ β‐adrenergic agonist drugs may cause weight loss by increasing energy expenditure and lipid breakdown.^46^ Conversely, several recent studies found that the use of glucocorticoids in treating allergic asthma induced and exacerbated obesity.^47^, ^48^ Differing associations between asthma medication and obesity in children may be due to the different physiological mechanisms of specific drug types and their targets.

There does not appear to be a common genetic link explaining asthma and obesity comorbidity in children. A study on the UK Biobank examining obesity‐related traits and asthma subtypes found no evidence for a genetic correlation between obesity and asthma that started before age 16 years.^49^

### 3.2 Asthma and depression and anxiety

A bidirectional relationship between asthma and mental health issues such as depression and anxiety has long been observed in adults,^50^, ^51^ and it is increasingly recognized that these relationships also extend to children with asthma.

**FIGURE 3:**
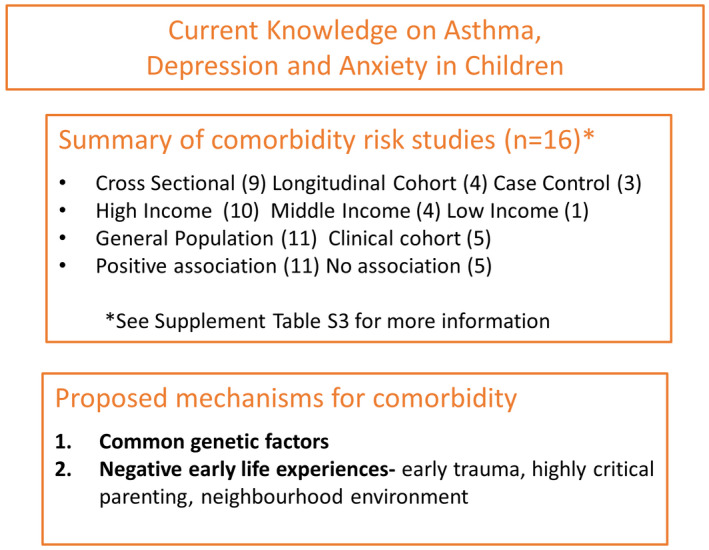
Current knowledge on asthma, depression and anxiety in children. Summary of study factors measuring depression/anxiety as a risk of asthma, and a summary of currently proposed mechanisms explaining the comorbidity of asthma and depression/anxiety.

Our search criteria found 32 relevant articles, 16 of which measured the association between paediatric asthma and depression/anxiety in a variety of countries and population groups from hospital‐based participants to large nationwide register studies (Table S3). The majority of studies (*n* = 13) found a positive association. The largest studies were a children's survey in South Korea (*n* = 788,411),^52^ which found an increased risk of depression and suicide ideation in adolescents with asthma (OR 1.12, 95% CI 1.09, 1.22 and OR 1.18, 95% CI 1.07, 1.24 respectively), and a register‐based study in Sweden (*n* = 281, 476),^53^ which found that children (ages 3–18 years) with asthma were more likely to have anxiety and other affective disorders (OR 3.04 and 2.50, respectively, 95% CI not provided). Similarly, a recent meta‐analysis confirmed an association of anxiety disorders in youth with asthma compared to youth without asthma (standardized mean difference 0.37, 95% CI 0.24, 0.50), Figure 3.^54^

A number of studies have shown that among children with asthma, those comorbid for depression and/or anxiety have increased risks of exacerbations,^55^, ^56^ poorly controlled asthma^57^, ^58^, ^59^, ^60^, ^61^ and increased medical costs including hospital stays^56^, ^62^, ^63^ compared to children without comorbidity. There is also some evidence to suggest that these risks are greater in girls than boys,^59^, ^61^, ^62^, ^64^ and in adolescents than younger children.^62^, ^65^ From a clinical perspective, this means that adolescent girls should be monitored carefully for depression and anxiety comorbidities, especially in regards to their asthma management.

There are several proposed mechanisms for asthma and anxiety/depression comorbidity – (1) genetic and/or (2) early environmental influences. The Childhood and Twin Study in Sweden (CATSS) of 9‐year‐old twins found evidence of familial co‐aggregation for asthma and depression/anxiety, that is, if one twin had asthma, the other twin was liable to have anxiety/depression regardless of the first twin's anxiety/depression status.^66^ This suggests that there is a common factor, either genetic or shared environment, that may explain clustering in families rather than a causal relationship between the diseases.

Recent developments in genome‐wide association studies have meant that the associations between the genetic liability for one disease and the risk of the symptoms of another disease can now be assessed. A population‐based cohort study on 16,687 children in Denmark aged 5–15 years found that a greater genetic liability for major depression measured as a polygenic risk score, was associated with an increased likelihood of having asthma (diagnosis or two medications in 12 months) and HR 1.06 (95% CI 1.01, 1.10) for each standard deviation in polygenic risk score increase.^67^ Similarly, in adults, a genetic correlation has been shown between depression and asthma using UK Biobank data (r_g_ = .17, *p* = .006).^68^ Possible genetic candidates have been suggested. In Bayesian network analysis, the top 100 single‐nucleotide polymorphisms (SNPs) for asthma symptom severity were obtained. From these, a single SNP rs4672619 was identified which interacts with depression to affect asthma symptom severity.^69^ This SNP is on the ERBB4 gene which is known to have a role in the pathophysiology of schizophrenia and bipolar depression and possible associations with childhood asthma.^69^ Other proposed genetic candidates include glucocorticoid receptor (GR) and beta2‐adrenergic receptor (β2‐AR) genes. In families with high levels of parental depression, youths with asthma have been shown to express significantly less GR and β2‐AR when they experience negative mood symptoms.^70^

In regard to early environmental influences, several recent parenting studies have shown that highly critical and over‐protective parenting may explain the higher level of anxiety in children with asthma.^71^, ^72^ However, these studies have been carried out in small parent–child dyad populations and require further research. Following a suite of studies over the previous decade in the USA on the role of neighbourhood and disadvantages on the risk of childhood asthma, Tobin et al have taken this research one step further to show that the relationship between neighbourhood stress and asthma in youth can be partly explained by anhedonia, a symptom of depression and the lack of experiencing pleasure.^73^ This theory of early trauma and neighbourhood environment as a trigger for comorbidity is supported by research on the World Trade Centre 9/11 terrorist attack.^61^ Children who lived in the neighbourhood of the World Trade Centre during 9/11 had high rates of poorly controlled asthma 10 years after the attacks (up to one‐quarter of children with asthma), which was associated with a higher risk of mental illnesses (OR 5.0, 95% CI 1.4, 17.7).^61^

### 3.3 Asthma and neurodevelopmental disorders

The prevalence of common childhood neurodevelopmental disorders such as attention deficit hyperactivity disorder (ADHD) and autism spectrum disorder (ASD) has increased during the last few decades.^74^, ^75^ The corresponding increasing comorbid conditions of asthma with neurodevelopmental disorders have a more substantial impact on children, carers and society than asthma alone.^76^ Despite this fact, clinical guidelines for asthma or neurodevelopmental disorders often overlook the existence of possible comorbidities.^77^ One particular challenge for children with concurrent asthma and ADHD and their parents is adequate disease management, such as appropriate use of inhalers, as well as stress and anxiety management to avoid asthma exacerbations.^78^

**FIGURE 4:**
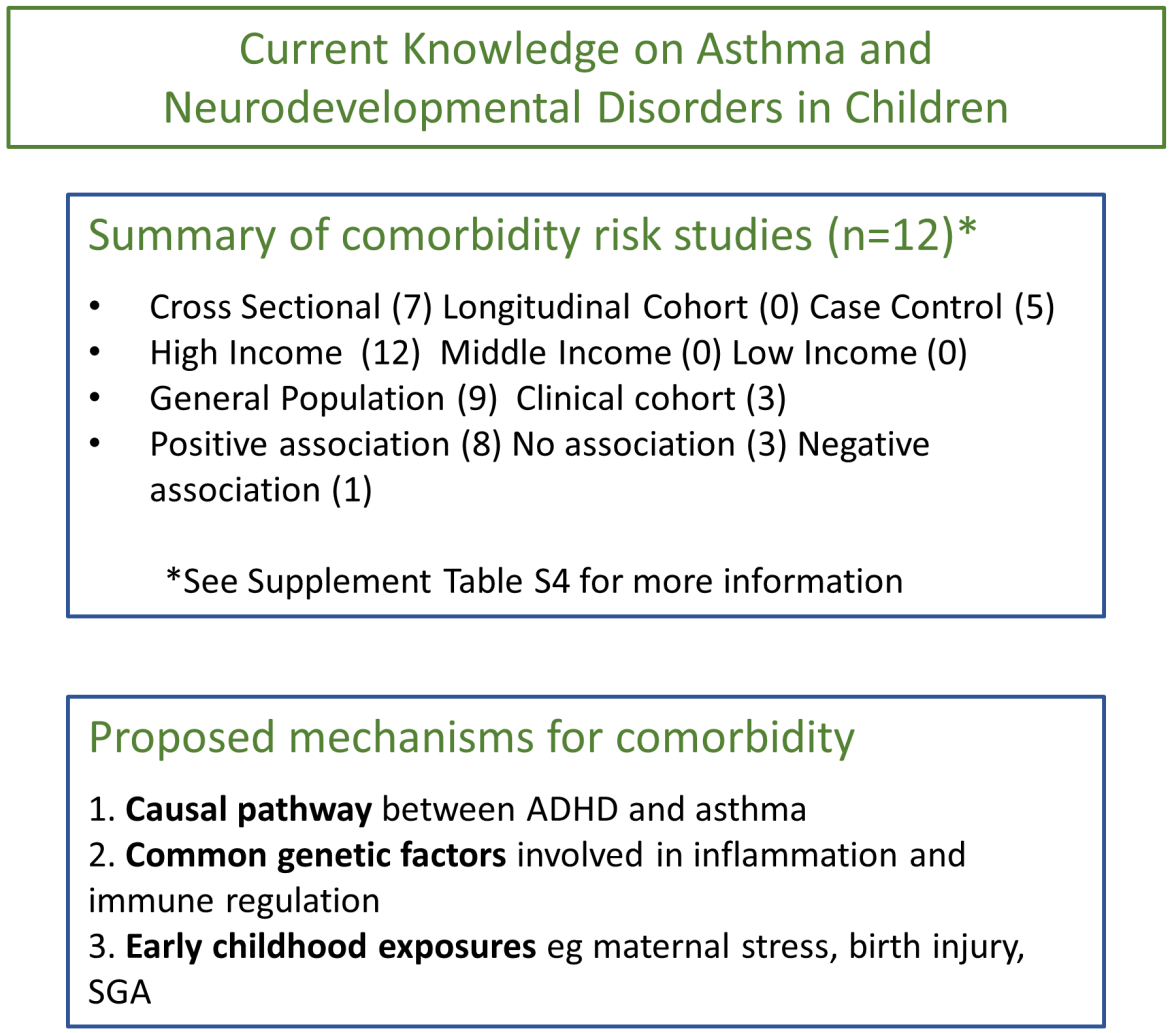
Current knowledge on asthma and neurodevelopmental disorders in children. Summary of study factors measuring neurodevelopmental disorders as a risk of asthma, and a summary of currently proposed mechanisms explaining the comorbidity of asthma and neurodevelopmental disorders.

Our search identified 23 relevant studies, of these, 12 estimated the association of asthma and neurodevelopmental disorders among children and adolescents from diverse ethnic backgrounds and in different settings (see Table S4). The majority of studies investigating the link between asthma and ADHD found positive associations,^79^, ^80^, ^81^, ^82^, ^83^, ^84^, ^85^, ^86^ most notably, two studies in Germany and Taiwan with over a million participants found associations of OR 2.19 (95%CI 2.16, 2.22)^85^ and OR 1.53 (95%CI 1.44, 1.63)^83^ respectively. Similarly, two recent meta‐analyses confirmed an association between asthma and ADHD (pooled OR 1.66, 95% CI 1.22, 2.26), and even suggested atopic diseases including asthma play a role in the severity of ADHD symptoms, Figure 4.^87^, ^88^

Regarding asthma and ASD, two of four studies found positive associations,^80^, ^89^ one found a null association^90^ and another reported a negative association.^91^ These last two studies were both clinically based samples of children recruited from hospital clinics, whereas the studies with positive associations used general populations. Of note, the largest study on ASD and asthma comorbidity from the National Survey of Children's Health in the USA (*n* = 71,084) found the risk for ASD was OR 1.68 (95% CI 1.07, 2.63) for children with asthma aged 0–17 years.^80^

Research on the aetiology of comorbidity between asthma and neurodevelopmental disorders occurring in children and adolescents suggests two possible major mechanisms: (1) shared risk factors via genetic and/or environmental factors, and (2) causation.

Firstly, evidence for a shared familial risk of ASD or ADHD with asthma has been identified in several family design studies.^80^, ^89^, ^92^, ^93^, ^94^ For example, Sun et al. using Swedish data found an increased risk of developing ADHD among relatives of individuals with asthma, and that the strength of the association was directly related to an increasing degree of genetic relatedness.^92^ Further decomposition of the familial liability among 11,547 twin pairs showed evidence for shared genetic factors explaining comorbidity.^92^ A large‐scale genome‐wide cross‐trait study using the UK Biobank and Psychiatric Genomics Consortium data confirmed a genetic correlation between ADHD with asthma and seven shared loci (highlighting the human leukocyte antigen [HLA] region), but no similar association was found for ASD with asthma.^95^ However, in vitro gene expression studies using tissues from patients with asthma and ASD have found gene polymorphisms close to the identified loci observed in the previous study (i.e. cyclooxygenase‐2 (COX‐2), adrenoceptor beta 2 (ADBR2) and GATA‐binding protein 3 (GATA3)) which are involved in inflammation and immune regulation. These may provide an insight into a genetic predisposition for asthma and ASD.^96^, ^97^, ^98^

Apart from shared genetic factors, shared environmental and lifestyle‐related factors, especially during the prenatal and perinatal periods, could provide an explanation for asthma and neurodevelopmental disorder comorbidity. There are a number of recent studies that suggest similar early risk factors for asthma and neurodevelopmental outcomes when studied separately but none have studied both diseases together. Such factors include maternal stress,^99^, ^100^ foetal distress, birth injury or trauma or small for gestational age.^101^, ^102^

Secondly, there is some evidence of direct causality between asthma and neurodevelopmental disorders. Zhu et al. provided insight into the pathological mechanisms of asthma with a Mendelian randomization approach, showing that ADHD may cause asthma but ASD probably does not.^95^ Furthermore, it is worthwhile to note that although the authors did not observe a causal pathway in the other direction from asthma to ADHD, there may be alternative explanations such as mediation by suboptimal asthma management, or that asthma is only causal for some ADHD phenotypes, for example, asthma predicts hyperactivity–impulsivity in adolescents but not inattention.^103^

Finally, most of the studies identified for this review reported the relationship between asthma and ADHD but did not consider the history of other atopic diseases as confounding factors. For example, Schmitt et al. observed that the association between eczema and ADHD remained after adjusting for asthma suggesting that eczema may play an important role in asthma and ADHD comorbidity.^104^

### 3.4 Asthma and sleep disorders

“Sleep disorders” is an umbrella term that includes several aspects of sleep including sleep quality/satisfaction/duration, daytime sleepiness, circadian rhythm and sleep disordered breathing (SDB). The prevalence of sleep disorders depends on which aspect is studied, but on the whole, sleep disorders are generally described as common in childhood.^105^, ^106^ Exploring the relationship between asthma and sleep disorders has been of interest for decades, in particular the role of asthma severity and asthma control as sleep can be disturbed by nocturnal asthma symptoms.^106^

**FIGURE 5:**
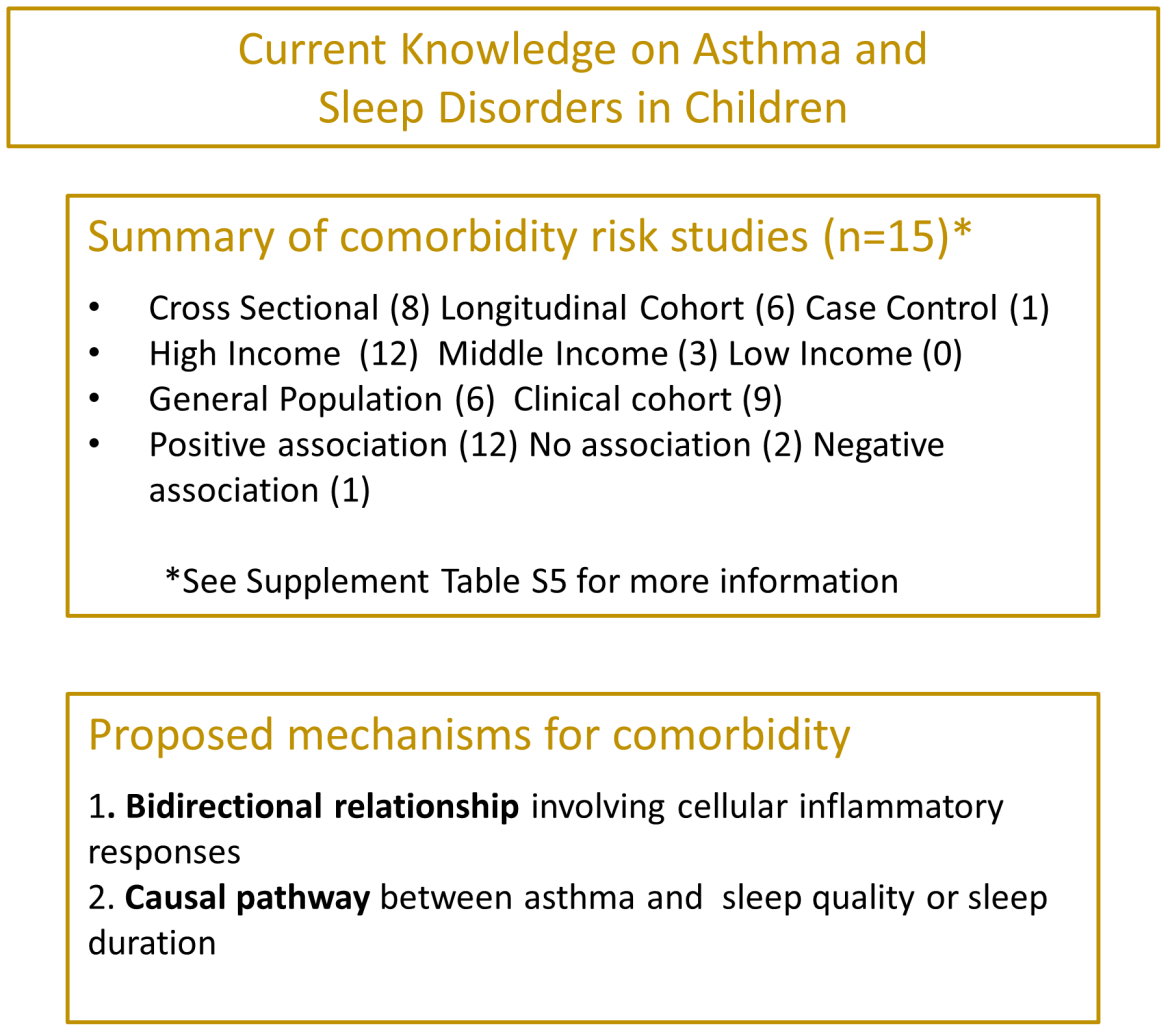
Current knowledge on asthma and sleep disorders in children. Summary of study factors measuring sleep disorders as a risk of asthma, and a summary of currently proposed mechanisms explaining the comorbidity of asthma and sleep disorders.

Our search revealed 23 studies, of which 15 studies investigated the strength of the comorbid association between asthma and various sleep disorders in children (Table S5); however, very little has been published on possible mechanisms, Figure 5.

Studies on sleep quality found that parents of children with asthma rated their child's sleep quality as poorer than those without asthma (χ^2^ = 27.07, *p* < .001), and children with asthma have reported poor sleep satisfaction (*p* < .001).^106^, ^107^ Some studies have suggested that poor sleep satisfaction may be explained by asthma severity and/or poor asthma control which are associated with lower sleep efficiency and sleep quality.^108^, ^109^, ^110^ This is supported by lung function studies in children with asthma which found that changes in spirometry measures of FEV_1_ (forced expiratory volume in 1 s) between night‐time and morning explained some of the variances of poorer sleep efficiency (13%, *p* < .1).^111^ However it should be noted that not all studies attribute sleep quality issues to asthma severity; one longitudinal study from Australia noted an association between asthma and sleep disturbance independent of asthma severity.^112^

Furthermore, two large population studies from South Korea and Brazil observed a negative association between asthma and sleep duration.^107^, ^113^ However, this association did not extend to studies on asthma severity.^109^, ^114^ In regards to sleep hygiene and daytime sleepiness, an association between poor asthma control with later bedtime and increased daytime sleepiness has been demonstrated,^106^, ^110^ but again, there was no association observed when focusing on asthma severity or asthma controller medication use.^109^

More convincing is the relationship between asthma and SDB. SDB encompasses a spectrum of disorders ranging from snoring to obstructive sleep apnoea (OSA) – a partial or complete cessation of airflow and oxygen desaturation during sleep despite the presence of breathing effort.^115^, ^116^, ^117^ Diagnosis and degree of severity of SBD are often measured using the Paediatric Sleep Questionnaire (PSQ), where a score of ≥0.33 suggests high risk of SDB. Adenotonsillar hypertrophy and obesity are significant risk factors for SDB, obesity has been shown to nearly double the risk of OSA.^118^ A systematic review from 2016 has demonstrated a bidirectional relationship between asthma and SDB.^119^ These findings are supported by Zandieh et al. in a large study of high school students (*n* = 9565) who showed that adolescents with asthma had 2.63 higher odds of reporting SDB‐like symptoms (95% CI 2.30, 3.00), and by Guo et al., OR 1.92 (95% CI 1.27, 2.91).^115^, ^120^ Furthermore, studies have found an inverse relationship between PSQ score and Asthma Control Test (ACT) score (*p* < .001),^121^, ^122^, ^123^ suggesting an association between SDB and poor asthma control. It has been proposed that the bidirectional association between asthma and SBD is due to inflammation in the “united airway,” for example, those with uncontrolled asthma have been found to have higher levels of tonsil TNF‐α compared to children with well‐controlled asthma.^105^, ^124^

Asthma is associated with both tonsillar hypertrophy and snoring, and although having an adenotonsillectomy improves asthma control,^112^, ^119^ in children with severe OSA, asthma increases the likelihood of needing treatment with continuous positive airway pressure after adenotonsillectomy, OR 2.78 (95% CI 1.36, 5.69).^125^ It is important to mention that the comorbidity between OSA and asthma is unclear when OSA is measured using the apnoea–hypopnoea index. Nguyen‐Hoang et al. have demonstrated an association between moderate asthma and severe OSA,^117^, ^119^ however, there are other studies showing a null or even negative association between asthma and OSA.^116^, ^118^, ^126^, ^127^ The ages of the study participants and the definitions of asthma varied between these studies, which may account for the observed differences in associations.

Owing to the physiological effects of obesity in the airway, and asthma and obesity both being associated with SDB, several studies have explored the relationship among asthma, obesity and SDB. Although, results are not as expected and indicate there is more to explore in this area. Andersen et al. found that BMI was positively associated with OSA independently of asthma^128^ and Narayanan et al. found that having asthma reduced the risk of severe OSA by 13.7% among obese patients.^127^

### 3.5 Asthma and autoimmune diseases

The relationship between asthma and autoimmune disease has long been debated. Whereas asthma, like other atopic diseases, is characterized by a Th2‐helper cell inflammatory response, autoimmune diseases are in general Th1 mediated. This dichotomy has remained a paradigm in the field, postulating that the two disease groups are mutually inhibitory. However, this notion has been questioned, both due to a deepened understanding of the complexities of the immune system but also because of epidemiological observations of co‐occurrence of asthma with autoimmune diseases.^129^, ^130^

**FIGURE 6:**
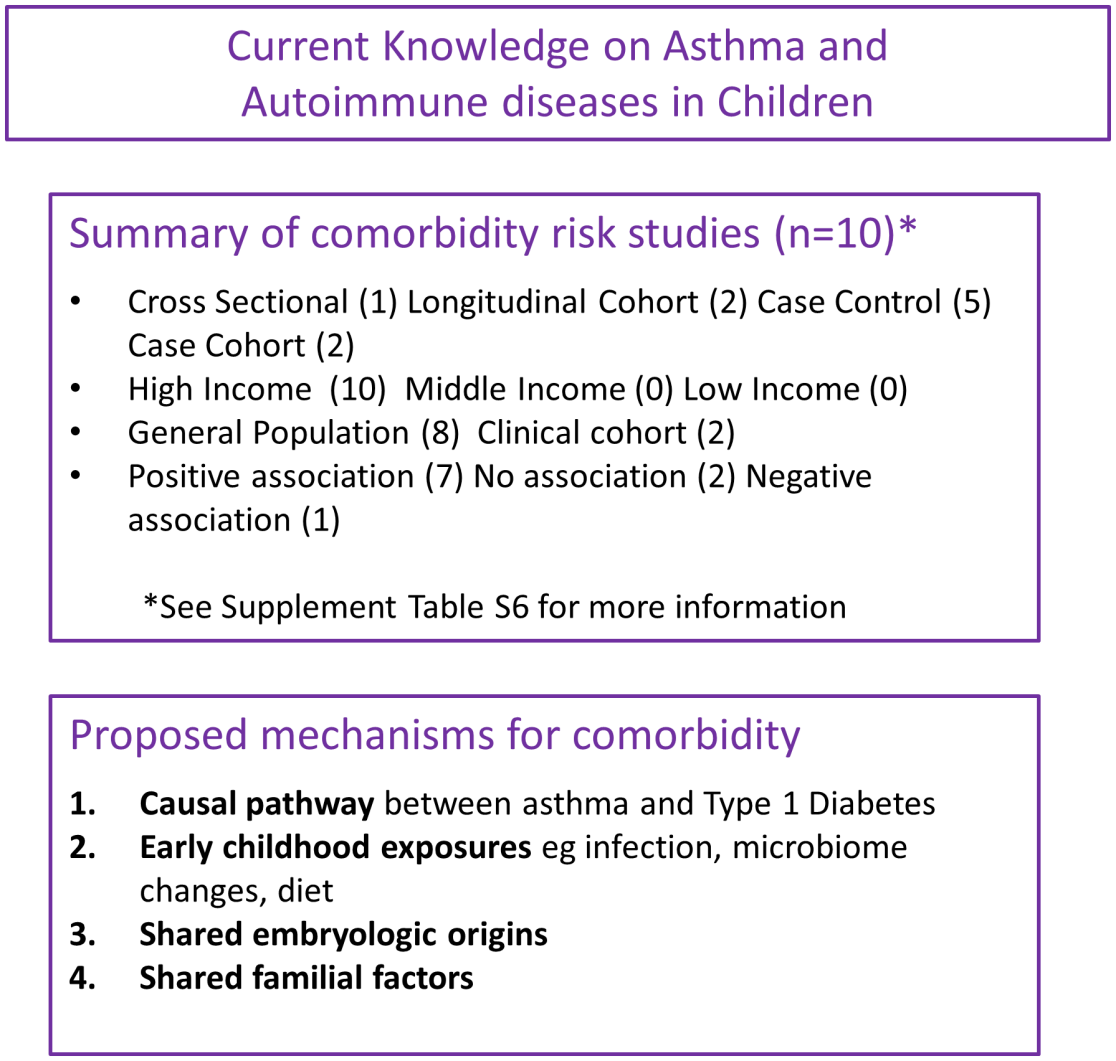
Current knowledge on asthma and autoimmune diseases in children. Summary of study factors measuring autoimmune diseases as a risk of asthma, and a summary of currently proposed mechanisms explaining the comorbidity of asthma and autoimmune diseases.

In our search of the most recent literature, we found 13 relevant articles of which 10 articles reported the magnitude of various autoimmune disease comorbidities among children and adolescents with asthma (Table S6). The panorama of autoimmune diseases reflects the most common types found in children such as celiac disease as well as less common but important types that entail a considerable burden on the affected such as type 1 diabetes (T1D) and inflammatory bowel disease (IBD). In general, the studies aimed to investigate the comorbidity between asthma and a named autoimmune disease, rather than to study the mechanisms underlying the relationship. Nevertheless, some studies have addressed the temporal relationship between the diseases which could shed light on causal mechanisms, Figure 6.

#### 3.5.1 Type 1 Diabetes

Despite inconclusive results of previous research regarding the potential comorbidity between asthma and T1D,^131^ more recent updates in the field have consolidated findings of co‐occurrence of the diseases.^130^ This includes two large population‐based studies from Finland and Sweden that demonstrated that asthma and T1D are often associated (HR 1.45, 95% CI 1.32, 1.60 and OR 1.15, 95% CI 1.06, 1.27 respectively).^132^, ^133^ Furthermore, the order of appearance of the respective diseases also seems to be of importance. In both studies, a previous diagnosis of asthma increased the risk of subsequent T1D (HR 1.45, 95% CI 1.32, 1.60,^130^ and HR 1.17, 95% CI 1.07, 1.28).^131^ However, the risk of asthma was reduced^132^ or unchanged^133^ among those who first developed T1D. Other evidence suggests that the potential causal influence of asthma on T1D could be mediated by asthma medication.^134^ Children using inhaled corticosteroids or beta‐agonists have been shown to be at increased risk of developing T1D even when adjusting for asthma status (HR 1.29, 95% CI 1.09, 1.52 and HR 1.22, 95% CI 1.07, 1.41 respectively).^135^

Familial co‐aggregation of asthma and T1D among siblings indicates that there are common risk factors for both diseases shared within families, either genetic or environmental.^133^ It is also important to acknowledge the potential existence of common risk factors that may not be shared within families but that could independently predispose individuals to both asthma and T1D. For instance, infections, changes to the microbiome and diet have all been separately linked to both asthma and T1D.^130^

#### 3.5.2 Inflammatory bowel disease

There are only a few recent studies on asthma comorbidity with IBD in children. In a Canadian population‐based study, Kuenzig et al. reported an association between asthma and IBD (OR 1.45, 95% CI 1.31, 1.60 for Crohn's Disease and OR 1.49, 95% CI 1.08, 2.07 for ulcerative colitis).^136^ A systematic review of the relationship between asthma and IBD in adults and children also found an overall association between asthma and IBD.^137^ However, subgrouping of the few studies that assessed paediatric‐onset IBD resulted in larger confidence intervals crossing the null: Crohn's disease (5 studies) pooled RR 1.35 (95% CI 0.94, 1.93) and ulcerative colitis (four studies) pooled RR 1.11 (95% CI 0.97, 1.28).^137^ More studies are needed to confirm or negate the comorbidity of asthma and IBD in children. The authors hypothesize that a potential explanation for comorbidity could be the shared embryologic origin of the airways and gut, or shared risk factors. It is, however, still unclear if there may be a causal association between the two diseases as well.

#### 3.5.3 Celiac disease, juvenile infantile arthritis, psoriasis and multiple sclerosis

Despite the celiac disease being the most common autoimmune disease in children, only one small study in our search investigated its association with asthma.^138^ The researchers found a non‐significant risk of celiac disease in children with doctor‐diagnosed asthma (OR 1.4, 95% CI 0.8, 2.5) that increased when applying an asthma definition based on family history of asthma (Asthma Predictive Index OR 2.8, 95% CI 1.3, 6.0), concluding that differences in asthma comorbidity risks may reflect different asthma phenotypes. Juvenile idiopathic arthritis^139^ and psoriasis^140^ were also found to be comorbid with asthma in single studies, but not multiple sclerosis,^141^ which is in line with findings in adults.^142^

## 4 COMMENTS AND CONCLUSION

In this review, we provide an up‐to‐date summary of the last 5 years of publications on non‐allergic comorbidities in children with asthma. Our goal was to provide a contemporary insight for magnitude and mechanisms that can be applied to a current paediatric audience and to inform those working clinically and in asthma research on the next steps in this field. The restriction of 5 years excludes many papers on all the comorbidities. However, we have included reviews where appropriate and we have found that the reported magnitudes confirm and strengthen previous findings that children with asthma have an increased risk of comorbid obesity, anxiety/depression, neurodevelopmental disorders, sleep disorders and autoimmune diseases.

Several of the studies we reviewed were large, population‐based and longitudinal with prospectively collected data and adequate control of possible confounding pathways. Others were cross‐sectional, relied on single measures of disease, failed to account for the difference in risk factors between early and later childhood and may be biased due to healthcare contacts.

Larger Genome Wide Association Studies (GWAS)‐based analyses, such as polygenic risk score prediction, expression, functional as well as pathway analyses, need to be carried out to further specify the most probable genes and how they could be involved in common pathways or causally linked. In addition, more detailed gene–environment interaction studies that include omics data and focus on early life exposures may help to clarify pathways of comorbidity at different points in the early life course.^143^ Asthma is commonly an outpatient diagnosis, and definitions of asthma tend to vary between studies; parent‐reported, self‐reported or physician diagnosed and, in very young children, recurrent wheeze as a proxy for asthma. These varying definitions may lead to overdiagnosis or misclassification of disease. As such, objective data on lung function, biomarkers and clinical measures including polygraphy and questionnaire data as well as timing to address causality will be valuable going forward. Asthma is a heterogenous disease, and although there is some research in children to suggest that comorbidities vary with phenotypes and endotypes, more research into this area has the potential to shed light on specific immunological mechanisms for comorbidity.

In addition to informing healthcare practice and asthma management guidelines, further understanding of the mechanisms for comorbidity can lead to targeted intervention studies for comorbidities, for example, cognitive behaviour‐based therapy has been shown to improve anxiety‐induced asthma.^144^, ^145^

In conclusion, further research is warranted to understand the comorbidity with a range of diseases as well as to better understand the underlying mechanisms in general. Clinical consequences of comorbidity in terms of management, severity/complications, medication, hospitalizations, healthcare cost and quality of life for individuals and families are of greatest importance.

## AUTHOR CONTRIBUTIONS

BB & CA conceived of the idea. All authors contributed to the analysis plan, search strategy, interpretation of data and approved the manuscript for submission. BB, ECO, TG, AH, MM, AS and CA sorted and collated the relevant papers from the literature search and wrote the first draft of the manuscript including the Supplementary Tables and creation of Figures.

## CONFLICT OF INTEREST

Jonas Ludvigsson has coordinated an unrelated study on behalf of the Swedish IBD quality register (SWIBREG), which received funding from Janssen corporation. Henrik Larsson reports receiving grants from Shire Pharmaceuticals; personal fees from and serving as a speaker for Medice, Shire/Takeda Pharmaceuticals, and Evolan Pharma AB; and sponsorship for a conference on attention‐deficit/hyperactivity disorder from Shire/Takeda Pharmaceuticals and Evolan Pharma AB, all outside the submitted work.

## Supporting information


Appendix S1

## Data Availability

Data sharing not applicable to this article as no datasets were generated or analysed during the current study.
