# Supplementary material for: Paediatric asthma and non‐allergic comorbidities: A review of current risk and proposed mechanisms
Source: Clin Exp Allergy. 2022 Jul 28;52(9):1035–47. doi: 10.1111/cea.14207 (PMC9541883; doi:10.1111/cea.14207)
Supplement: Supplementary file 1 — Appendix S1 [file CEA-52-1035-s001.docx]

## Supplement – Asthma comorbidity review

**Table S1. Search terms used for Comorbidities**

| **Autoimmune diseases** | Exp Celiac Disease/  Exp Diabetes Mellitus, Type 1/  Exp Inflammatory Bowel Diseases/  Exp arthritis, Rheumatoid/  Exp Arthritis, Juvenile/  Exp Crohn Disease/  Exp Hypothyroidism/  Exp psoriasis/  Exp Sarcoidosis/  Exp Lupus Erythematosus, systemic/  Exp Colitis, Ulcerative/  Exp Purpura, Thrombocytopenic, Idiopathic/  arthriti* or still disease* or polyarthriti* or oligoarthriti* or gluten* enteropathy or (sprue adj2 celiac*) or type 1 diabetes or diabetes type 1 or autoimmun* diabet* or inflammatory bowel disease* or bowel disease* inflammatory* or psoriasis* or crohns disease* or hypothyroidism* or sarcoidosis* or systemic lupus erythematosus or ulcerative colitis or multiple sclerosis or psoriasis or idiopathic thrombocytopenia or immune thrombocytopenia |
| --- | --- |
| **Anxiety and Depression** | Exp Anxiety/  Exp Depression/  Anxious* or anxiety* or depression* or depressive* or mental health or psychiatric disorder* |
| **Neurodevelopmental disorders** | Exp Neurodevelopmental disorders/  ((attention deficit or neurodevelopmental) adj3 disorder*) or (adhd or autism* or autistic) |
| **Obesity and Overweight** | Exp overweight  Overweight or obes* |
| **Sleep disorders** | Exp Sleep Wake Disorders/  apnea* or sleep disorder or sleep disturbance* or sleep disordered breathing or sleep problem* or sleep disruption* or sleep hygiene |
|  |  |

List of abbreviations: ACT (asthma control test), ASD (autism spectrum disorder), ADHD (attention deficit hyperactivity disorder), AHI (apnea-hypopnea index), API (asthma predictive index), BMI (body mass index), CI (confidence interval), GINA(Global Initiative for Asthma) HR (hazard ratio), IBD (inflammatory bowel disease), ICD (International Classification of Disease code), ISAAC (International Study of Asthma and Allergies in Childhood), (OR (odds ratio), OSA (obstructive sleep apnea), PD (prevalence difference), PSQ (Pediatric Sleep Questionnaire), (RR (relative risk), SDB (sleep disordered breathing), SDQ (Strengths and Difficulties Questionnaire), SNP (single nucleotide polymorphism), T1D (Type 1 diabetes mellitus)

**Table S2. Asthma in children and the risk of Obesity (Studies 2016-2021)**

| **First authors & year** | **Country & Study name** | **Study design** | **Population type & size** | **Age of children (yrs)** | **Asthma definition** | **Obesity definition** | **Association between asthma and obesity** |
| --- | --- | --- | --- | --- | --- | --- | --- |
| **Alvarez Zallo 2017^1^** | Spain.  ISAAC phase III. | Cross-sectional multi-center study | General Population  N= 8,607 | 6-7 &  13-14 | Ever had asthma (parent report) | BMI cut-off points set by Cole et al ^2^ for each group by age and sex. | 6-7 years:  OR 2.29 (95%CI 1.43, 3.68)  13-14 years:  OR 1.18 (95%CI 0.41-3.43) |
| **Chen 2017^3^** | USA | Cohort | Children from schools in eight communities, n=2171 | 5-8 | Ever had asthma (parent report)  Lifetime asthma and wheeze during the previous year (parent report) | Age- and sex-specific BMI ≥95th percentile (Centers for Disease Control and Prevention) | HR: 1.51 (95%CI 1.08,2.09) |
| **Contreras 2018^4^** | 16 European countries -Mechanisms of the Development of Allergy (MeDALL)  consortium & the Child Cohort Research Strategy for Europe (CHICOS) | Multicentre Birth cohorts | 16 European birth cohorts  n= 21,130 | 4.1 | ISAAC- Doctor diagnosis (parent report) | Defined according to the 2012 Cole-International Obesity Task Force, age and sex-specific cut-off. | HR 1.66 (95%CI 1.18, 2.33) |
| **den Dekker 2017^5^** | Netherlands, Generation R Study | Prospective cohort | General population  n = 6,178 | 6 | ISAAC- Doctor diagnosis (parent report) | Z scores using known prevalence (2.9%) of obesity in children aged 6 years in The Netherlands. | RR 1.20 (95%CI 0.67, 2.14) |
| **Joseph 2016^6^** | USA  National Survey of Children’s Health | Cross-sectional | General population  n =88,668 | 10-17 | Doctor diagnosis (parent report) | Age- and sex-specific BMI ≥95th percentile | RR 1.50 (95%CI 1.45, 1.56) |
| **Lai 2020^7^** | China | Cross-sectional | General population, 13 primary schools  n= 16,837 | 6-12 | Doctor diagnosis (parent report) | BMI z-score was calculated by age- and sex-specific BMI cutoffs (World Health Organization) | OR 1.51 (95%CI 1.03, 2.21) |
| **Sullivan 2020^8^** | USA.  Medical Expenditure Panel Survey. | Cross-sectional | General population n=50,666 | 6-17 | Combination of survey questions common to the National Health Interview Study (NHIS) about current and lifetime asthma in combination with healthcare utilization with ICD-9 493 | Based on national age‐adjusted BMI percentiles (Centers for Disease and Control and Prevention) | RR 1.67 (95%CI 1.32, 2.11) |
| **Vezir 2021^9^** | Turkey | Age- and sex-matched groups | Patients in outpatient clinics, n=153 | 6-18 | Diagnosis of asthma  ≥1 year (GINA) and/or having asthma symptoms ≥1 year. | Age- and sex-specific BMI ≥95th percentile | RR 1.04 (95%CI 0.75, 1.45) |
| **Zhang 2019^10^** | USA.  Southern California Childrens Health Study | Multi-cohort | General population, 13 communities  n = 5,193 | 5-7 | Doctor diagnosis (parent report) | Age- and sex-specific BMI ≥95th percentile (Centers for Disease Control and Prevention) | OR 1.38 (95%CI 1.12, 1.71) |

*Italics indicates the asthma definition used in the risk prediction*

**Table S3. Asthma in children and the risk of Anxiety and/or Depression (studies 2016-2021)**

| **First author & year** | **Country & study name** | **Study design** | **Population type & size** | **Age of children (yrs)** | **Asthma definition** | **Anxiety/depression**  **definition** | **Association between asthma and anxiety/depression** |
| --- | --- | --- | --- | --- | --- | --- | --- |
| **Agnafors 2019^11^** | Sweden | Cross-sectional | General population- all children from one region in Sweden, n=281,476 | 3-18 | Diagnosis J45/46 by specialist or primary care | Affective mood disorder diagnosis F30-F39, anxiety F40-F49 by specialist or primary care | **Anxiety**:  OR 3.04  **Affective disorders**:  OR 2.50 |
| **Ahmadiafshar^12^ 2016** | Iran | Cross-sectional | General population, randomly chosen from 20 schools, n=1500 | 13-14 | Doctor diagnosis, or ISAAC questions- dry cough> 2 weeks + night symptoms + wheeze not attributable to other diseases (self-report) | Beck Depression Inventory | **Depression**  OR 2.82 (95%CI 1.96, 4.21) |
| **Arif 2016^13^** | Pakistan | Cross-sectional | Patients from hospital outpatient clinic, n=400 | Ave 5.9 | Doctor diagnosis (primary care physician) | Doctor diagnosis of anxiety problems (parent report) | **Anxiety**  OR 13.5 (95%CI 7.3, 24.9) |
| **Barton 2020^14^** | Australia, MAC study | Birth Cohort | Family history of allergy, n=620 | 18 | Current doctor diagnosed and asthma episode /wheeze / use of asthma medication <12m (self-report) | Kessler scale- serious psychological distress | No association, p=0.86 |
| **Brew 2018^15^** | Sweden, CATSS | Cross-sectional | Twins from general population, n= 14 197 | 9 | Current asthma (parent report) or  algorithm based on hospital diagnosis and/or medication | Screen for Child Anxiety Related Disorders (anxiety)  Short Mood and Feelings Questionnaire (depression) | **Anxiety or Depression**  *Parent report asthma:*  OR 1.59 (95%CI 1.36, 1.96)  *Register based asthma*:  OR 1.31 (95%CI 1.09, 1.56) |
| **Dogru 2019^16^** | Turkey | Case control (not matched) | Convenience sampling from outpatient clinic, n=64 | 8-18 | Asthma diagnosis for at least one year | Kiddie Schedule for Affective Disorders and Schizophrenia:: semi structured interview form- generalised anxiety disorder (GAD) | **GAD**  OR 4.20 (95%CI 1.18, 14.94) |
| **Dut 2021^17^** | Turkey | Cross-sectional | n=171 | Ave 14.2 (SD 1.9) | Diagnosis of asthma for at least 6 months (GINA)  ACT | Brief Symptom Inventory (BSI) for anxiety and depression | **Depression**  p= 0.85  **Anxiety**  p=0.17 |
| **Edvinsson Sollander 2021^18^** | Sweden, Children and Parents in Focus Project | Cross-sectional | General population- recruited from child health centres, n=4649 | 3-5 | Doctor diagnosis (parent report) | Strengths and Difficulties Questionnaire (SDQ) (parent answered) and emotional problems (teacher answered) | **Emotional problems**  Rated by parents:  OR 1.34 (95%CI 1.02, 1.76)  Rated by teachers:  OR 1.44 (95%CI 1.09, 1.91) |
| **Hammer-Helmich 2016^19^** | Denmark | Cross-sectional | General population survey, n=9215 | 3-15 | Ever asthma (self-report) | SDQ- abnormal scores of emotional problems | **Emotional Problems**  OR 1.30 (95%CI 0.98, 1.72) |
| **Kim 2020^20^** | Korea, Korea Youth Risk Behaviour Web-Based Survey | Cross-sectional | National based survey,  n= 195 847 | 12-18 | Doctor diagnosis (self-report) | Suicide ideation (self-report), Depression measured with CIDI | **Suicide Ideation**  **Depression**  p<0.0001 |
| **Kyung 2021^21^** | Korea, Korea Youth Risk Behaviour Web-Based Survey | Longitudinal | National based survey  n=788 411 | 12-18 | Doctor diagnosis (self-report) | Stress, depression, suicide ideation (self-report) | **Depression**  OR 1.12 (95%CI 1.09, 1.22)  **Suicidal ideation**  OR 1.18 (95%CI 1.07, 1.24) |
| **Kimura 2020^22^** | Japan | Cross-sectional | General population cohort from one city, n=17 752 | 6-12 | Doctor diagnosis (parent report) | SDQ- anxiety | **Anxiety**  PR 1.39 (95%CI 1.15, 1.68) |
| **Laurens 2019^23^** | Australia, NSW-CDS study | Longitudinal | General population, NSW register based linkage, n= 21 302 | 5-11 | Diagnosis in hospital or emergency department | Australian Educational Development Census- age 5: anxious and fearful behaviour (teacher report)  MCS, based on SDQ: emotional symptoms (self-report) | **Anxiety**  Age 5:  OR 1.15 (95%CI 0.95, 1.38)  Age 11:  OR 1.12 (95%CI 0.93, 1.34) |
| **Mpairwe 2021^24^** | Uganda | Nested Case control | General population from schools in a single district,  n= 162 | 12-17 | ISAAC and clinical assessment | Y1-4R (youth Inventory) scale to measure GAD, panic disorder and social anxiety disorder | **Anxiety**  OR 2.68 (95%CI 1.20, 5.53) |
| **Rajhans 2021^25^** | India | Case control | Moderate to severe asthma patients, matched to healthy controls n=60 | 8-15 | GINA guidelines for moderate to severe asthma | Child Behaviour Checklist (CBCL): anxiety/depression, withdrawn/ depressed or internalizing behaviours | **Anxious/ depressed**: p=0.017  **Withdrawn/depressed/internalizing behaviours**:  p= NS |
| **Van der Laan 2021^26^** | Netherlands, PIAMA | Birth cohort | General population-recruited in pregnancy, n=2660 | 11-17 | 2 of 3 criteria:   - Doctor diagnosis - Wheeze <12 months - Asthma medication <12 months | Mental Health Index-5, mental wellbeing  (scale 0-100) | **Mental wellbeing**  No significant association |

*Italics indicates the asthma definition used in the risk prediction*

**Table S4. Asthma in children and risk of Neurodevelopmental Disorders (studies 2016-2021)**

| **First author & year** | **Country & Study name** | **Study design** | **Population type & size** | **Age of children (yrs)** | **Asthma definition** | **Neurodevelopmental disorder definition** | **Association between asthma and neurodevelopmental disorders** |
| --- | --- | --- | --- | --- | --- | --- | --- |
| **Akmatov 2021^27^** | Germany, nationwide claims database | Case-control | General population, national database.  n=2 586 620 | 5-14 | Doctor diagnosis by ICD code (register-based) | ADHD doctor diagnosis by ICD code (register-based) | **ADHD**  OR 2.19 (95%CI 2.16,2,22). |
| **Chai 2021^28^** | Canada | Cross-sectional & cohort | General population, n=768 460 | 0-22 | Doctor diagnosis by ICD code (register-based) | ADHD doctor diagnosis by ICD code (register-based) | **ADHD**  OR 9.87 (95%CI 8.59, 11.15) for males  OR 9.92 (95%CI 8.35, 11.49) for females |
| **Chen 2017^29^** | Taiwan | Case control | General population, randomly selected sample n= 1,000,000 | 0-17 | Doctor diagnosis by ICD code (register-based) | ADHD doctor diagnosis by ICD code (register-based) | **ADHD**  OR 1.53 (95%CI 1.44, 1.63) |
| **Dai 2019^30^** | Taiwan | Case control | General population, n=13 810 (2762 ASD cases, 11 048 matched controls | Ave 12.8 (SD 4.7) | Doctor diagnosis by ICD code (register-based) | ASD doctor diagnosis by ICD code (register-based) | **ASD**  RR 1.24 (95%CI 1.16, 1.33) |
| **Jameson**  **2016^31^** | US, National Comorbidity Survey | Cross-sectional | General population n=6483 | 13-18 | Parent report asthma | Lifetime ADHD if met 4 criteria: A- six symptoms of inattention and/or hyperactivity-impulsivity; B- symptoms causing impairment before 7 years; C- impairment in at least 2 settings (school/ work/ home); D- clinically significant impairment in social, academic or occupational functioning. | **Lifetime ADHD**  OR 0.94 (95%CI 0.76, 1.16). |
| **Jonsdottir 2017^32^** | US | Case-control & cohort | Clinical sample, n=1568 | 2-17 | Doctor diagnosis by ICD code (register-based) | ASD doctor diagnosis by ICD code (register-based) | **ASD**  OR 0.48 (95%CI 0.33, 0.71) |
| **Strom 2016^33^** | US, National Health Interview Survey (NHIS) & National Survey of Children’s Health (NSCH) | Cross-sectional | General population,  n=354 416 | 2-17 | History of asthma (parent report) | History of childhood ADD/ADHD (parent report) | **ADHD**  OR 1.91 (95%CI 1.83, 1.99). |
| **van der Schans 2016^34^** | Netherlands | Nested case-control | General population,  n=21 285 | 6-12 | ≥3 prescriptions of inhaled corticosteroid or short-acting β-mimetic in 12 months | ≥2 prescriptions of methylphenidate (ADHD medication) in 12 months | **ADHD**  OR 1.4 (95%CI 1.3, 1.6) |
| **Wang 2018^35^** | Taiwan | Cross-sectional | Survey/case-control, n=432 | 8-10 | ISAAC questions (asthma with sensitization) | ADHD: DSM-IV diagnosis | **ADHD**  OR 0.87 (95%CI 0.51, 1.31) |
| **Weber 2017^36^** | US | Cross-sectional | Hospital sample, n=1242 | 6-18 | Parent report asthma | ASD doctor diagnosis (clinician) | **ASD**  0.95 (95%CI 0.69, 1.31). |
| **Xie 2020^37^** | US, National Survey of Children’s Health (NSCH) | Cross-sectional | General population,  n = 71 084 | 0-17 | Doctor diagnosis and current symptoms (parent report) | ASD, ADHD, intellectual or learning disability (parent report) | **ADHD**  OR 2.01 (95%CI 1.67, 2.42)  **ASD**  OR 1.68 (95%CI 1.07, 2.63) |
| **Yang 2018^38^** | Taiwan | Cross-sectional | General population, children from 11 communities n= 2772 | 3-6 | Doctor diagnosis AND nocturnal cough or exercise-induced wheeze in last 12 months (parent report) | ADHD: DSM-IV diagnosis | **ADHD**  OR 4.00 (95%CI 1.44, 11.13). |

**Table S5. Asthma in children and risk of Sleep Disorders (Studies 2016 -2021)**

| **First author & year** | **Country & Study name** | **Study design** | **Population type & size** | **Age of children (yrs)** | **Asthma definition** | **Sleep Disorder**  **definition** | **Association of Asthma with Sleep Disorders** |
| --- | --- | --- | --- | --- | --- | --- | --- |
| **Andersen**  **2019^39^** | Denmark | Cross sectional | Obesity clinic and schools, n=172 | 7-18 | Asthma and asthma medication (self-report) | Obstructive sleep apnoea (OSA) based on Apnoea–Hypopnea Index (AHI) | **OSA**  OR 0.51 (95%CI 0.16, 1.69) |
| **Dooley 2020^40^** | USA | Retrospective cohort study | Paediatric asthma clinic,  n= 205 | 1-21 | Clinician diagnosed asthma and Severity graded by asthma control test (ACT) score | Sleep disordered breathing (SDB) based on Paediatric Sleep Questionnaire (PSQ) (parent report) | **Sleep disordered breathing** *ACT score*  p= 0.01 |
| **Estanislau 2020^41^** | Brazil  (ERICA) | Cross-sectional | School children  n= 59,442 | 12-17 | ISAAC questionnaire: at least one wheezing attack in the last 12 months (parent report) | Sleep duration based on questionnaire (parent report) | **Sleep duration**  PR 1.25 (95%CI 1.08, 1.44) |
| **Garden 2016^42^** | Australia (MUSP) | Cross-sectional | Birth cohort  n= 5015 | 14 | Asthma with symptoms ‘sometimes’ or ‘often’ in the last 6 months (parent report) | Sleep (quality and daytime somnolence) and snoring  (parent report) | **Sleep (quality and daytime somnolence)**  OR 1.45 (95%CI 1.19–1.75)  **Snoring**  OR 1.70 (95%CI 1.46–1.98) |
| **Guo 2020^43^** | China | Cross-sectional | School-based population,  n= 3997 | 3-14 | Doctor diagnosed (parent report) confirmed with clinician | Sleep disordered breathing measured with PSQ | **Sleep disordered breathing** OR 1.92 (95%CI 1.27, 2.91) |
| **Kilaikode 2019^44^** | USA | Cross sectional | Patients from pediatric sleep clinic, n= 400 | 0-20 | Doctor diagnosed (clinician) and using asthma medication. | Severe OSA based on AHI (≥10 events per hour) | **Need for CPAP with severe OSA**  OR 2.78 (95%CI 1.36, 5.69) |
| **Martin 2017^45^** | USA | Cohort | Participants from school and paediatric clinic, n=346 | 7–9 | Doctor diagnosed (clinician) | Sleep duration and sleep efficiency by actigraph. Sleep hygiene and daytime sleepiness by questionnaire | S**leep efficiency** and **daytime sleepiness**, p < 0.05 |
| **Meltzer 2017^46^** | USA | Cross sectional | General population, n=364 | 1-4 | Doctor diagnosis and/or multiple episodes of wheezing in last 12 months and/or prescribed quick relief medication for breathing problems (parent report) | Sleep quality based on questionnaire | S**leep quality**,  p<0.0001 |
| **Narayanan 2019^47^** | USA | Retrospective case control | Paediatric sleep clinic, n=367 | 9 - 17 | Doctor diagnosis identified from medical records | OSA based on AHI | **Severe OSA**  OR 0.55 (95%CI 0.34, 0.88) |
| **Nguyen-Hoang 2017^48^** | Vietnam | Prospective cohort study | Paediatric asthma clinic, n=85 | 6-12 | GINA guidelines | OSA based on AHI | **Severe OSA**  p<0.01 |
| **Perikleous 2018^49^** | Greece | Cross sectional | Paediatric asthma clinic, n=140 | 4 -12 | Doctor diagnosed (clinician) | SDB measured with PSQ | **Sleep Disordered Breathing** *ACT score*  r = -0.356, p < 0.001 |
| **Reynolds 2018^50^** | USA  (NAPS) | Cohort | Children with persistent asthma from Nocturnal Asthma and Performance in School (NAPS) study, n= 249 | 7-9 | Doctor diagnosed (clinician) | Sleep efficiency and sleep duration measured actigraphy | S**leep efficiency,**  p=0.024 |
| **Tamanyan 2016^51^** | Australia | Retrospective cohort study | Paediatric sleep clinic, n=301 | 3-17 | Parent report | OSA based on AHI | **OSA**  p=0.189 |
| **Zaffanello 2017^52^** | Switzerland | Retrospective cohort study | Paediatric sleep clinic, n=134 | 2-18 | Doctor diagnosis in medical records | OSA based on obstructive AHI | **OSA**  p=0.733 |
| **Zandieh 2017^53^** | USA | Cross sectional | High school students,  n= 9,565 | Ave 15.9 | Doctor diagnosis asthma, reactive airway disease or wheezy bronchitis (self-report) | Sleep disordered breathing (self-report) | **Sleep Disordered Breathing** OR 2.63 (95%CI 2.30,3.00) |

*Italics indicates the asthma definition used in the risk prediction*

**Table S6. Asthma in children and risk of Autoimmune Diseases (studies 2016-2021)**

| **First author & year** | **Country & study name** | **Study design** | **Population type & size** | **Age of children (yrs)** | **Asthma definition** | **Autoimmune disease definition** | **Association between asthma and autoimmune disease** |
| --- | --- | --- | --- | --- | --- | --- | --- |
| **Bourne 2017^54^** | USA, “The environment and genetic risk factors for pediatric MS study” | Case-control, retrospective questionnaire data | Total, n=689  Cases with MS and controls from 16 clinics included in a US MS network , n=271 ,418 | Median 15.7 (IQR 13.5,17.3) | Ever asthma (parent report) | **Multiple Sclerosis:**  Diagnosis< 18 yrs of age, disease for <4 yrs in US Network of Pediatric MS. | **Multiple Sclerosis:**  OR 1.04 (95%CI 0.66, 1.66) |
| **Ghersin 2020^55^** | Israel | Cross-sectional, case-control | Jewish adolescents who underwent general health evaluation prior to military enlistment, n=1,142,732, cases n=891 | Median 17.1, (IQR 16.7,17.3) | Diagnosis from medical history documents provided by family physician. | **Inflammatory Bowel Disease:** Gastroenterologist confirmed report: endoscopy, histology, radiology | **Crohn’s Disease**  p-value 0.16.  **Ulcerative Colitis**,  OR 0.46 (95%CI 0.21, 0.97) |
| **Kim 2019^56^** | South Korea, “Korean Health Insurance Review and Assessment Service-National Sample Cohort” | Matched cohort study | General population. Individuals with asthma, n=167,693. Controls matched 1:1,  n=165,288 | <15 | Medical claim codes, ICD-10 (J45 J46) AND > 3 treatments of Inhaled Corticosteroids (ICS), Long-Acting Bronchodilator inhalers (LABA), Leukotriene Receptor Antagonists (LRTA) | **Psoriasis:**  Diagnosis J02 + treatment more than three times | **Psoriasis**  HR 1.35 (95%CI 1.07, 1.69) |
| **Kuenzig 2017^57^** | Canada | Case control, register-based | Cases with IBD: Crohn’s Disease n=3087, Ulcerative Colitis =2377  Controls from general population enrolled in insurance plan, n=402,800 | 3-16 | Validated algorithm based on ICD-codes – 2 or more If ambulatory, 1 or more if from discharge records (register-based) | **Inflammatory Bowel Disease:** cases from validated register definitions (repeated IBD diagnoses) | **Crohn’s Disease**  OR 1.45 (95%CI 1.31, 1.60)  **Ulcerative Colitis**  OR 1.49 (95%CI 1.08, 2.07) |
| **Lin 2016^58^** | Taiwan | Case control, population-based | General population based on national health insurance claims.  JIA, n=167  Matched on sex 1:4 to controls, n=668 | Ave 11.3 (SD 3.5) | ICD-9 code 493, at least 3 times in inpatient, or ambulatory care for asthma (register-based) | **Juvenile Idiopathic Arthritis (JIA):**  ICD-9 codes for JIA <16y from database for diseases that require long-term care | **JIA**  All ages: OR 1.44 (95%CI 1.00, 2.10)  <12 yrs: OR 1.97 (95%CI 1.19, 3.25). |
| **Metsälä 2020^59^** | Finland | Case-cohort, population-based | Cases with T1D, n=3348. 10% random sample of general population as control. | Median 5.0 (IQR 2.9-7.7) | Anti-asthmatic drugs as exposure, ATC R07 and divided into further subgroups | **Type 1 Diabetes:**  Reimbursements for insulin prescription | **Type 1 Diabetes**  *Inhaled Corticosteroids*  HR 1.29 (95%CI 1.09, 1.52)  *Inhaled beta-agonists* HR 1.22 (95%CI 1.07, 1.41) |
| **Metsälä 2018^60^** | Finland | Case-cohort, population-based | General population, T1D cases n=9541 and asthma n=81,473 before 16 y. 10% random sample of general population as control, n=171,138 | Median 7.5 | Reimbursements for medication for chronic diseases, requires diagnosis verified by specialist and long-lasting drug treatment | **Type 1 Diabetes:**  Reimbursement records for insulin prescription, requires diagnosis. | **Type 1 Diabetes**  HR 1.45 (95%CI 1.32, 1.60). |
| **Patel 2018^61^** | USA  “Rochester Epidemiology Project (REP)” | Case-control  Population-based | Cases of children with celiac disease n=94. Controls matched on sex and birth year, n=188 (94 from REP, 94 who had undergone CD screening but tested negative) | Median 9.7 (IQR 6.5-13.1) | Review of medical records. Two independent criteria:  1. Predetermined asthma criteria including doctor diagnosis of asthma or symptoms  2. Asthma predictive index (API) | **Celiac disease:**  ICD-9 579.0. from medical linkage and verified with manual medical chart review (positive serology and confirmatory biopsy) | **Celiac disease:**  *Predetermined asthma criteria*  OR 1.4 (95%CI 0.8, 2.5)  *API*  OR 2.8 (95%CI 1.3, 6.0) |
| **Smew 2020^62^** | Sweden | Population-based register cohort study | General population, nationwide. n=1,284,748 | Ave  5.9 (SD 3.3) | Validated definition based on combination of asthma diagnosis and prescription of asthma medications (register-based) | **Type 1 Diabetes:**  insulin prescription and/or T1D diagnosis (register-based) | **Type 1 Diabetes:**  *Current asthma*  OR 1.15 (95%CI 1.06, 1.27)  *Previous asthma*  OR 1.17 (95%CI 1.07, 1.28). |
| **Wasielewska 2019^63^** | Poland | Case-control | Cases and controls from hospital pediatric unit. Cases of IBD, n=60 and controls, n=60 | Ave 14.7 (SD 3.4) | Questionnaire on allergic diseases, modified from the Global Initiative for Asthma and International Study of Asthma and Allergies in Childhood | **Inflammatory Bowel Disease:**  European Society guidelines – clinical, endoscopic, histopathological and radiological criteria | **Crohn’s disease**:  OR 0.49 (95%CI 0.15, 1.58)  **Ulcerative colitis**  OR 0.37 (95%CI 0.08, 1.71). |

*Italics indicates the asthma definition used in the risk prediction*

**REFERENCES**

1. Alvarez Zallo N, Aguinaga-Ontoso I, Alvarez-Alvarez I, Guillen-Grima F, Azcona San Julian C. The influence of gender and atopy in the relationship between obesity and asthma in childhood. *Allergologia et immunopathologia.* 2017;45(3):227-233.

2. Cole TJ, Bellizzi MC, Flegal KM, Dietz WH. Establishing a standard definition for child overweight and obesity worldwide: international survey. *BMJ (Clinical research ed).* 2000;320(7244):1240-1243.

3. Chen Z, Salam MT, Alderete TL, et al. Effects of Childhood Asthma on the Development of Obesity among School-aged Children. *Am J Resp Crit Care Med.* 2017;195(9):1181-1188.

4. Contreras ZA, Chen Z, Roumeliotaki T, et al. Does early onset asthma increase childhood obesity risk? A pooled analysis of 16 European cohorts. *Eur Resp J.* 2018;52(3).

5. den Dekker HT, Ros KPI, de Jongste JC, Reiss IK, Jaddoe VW, Duijts L. Body fat mass distribution and interrupter resistance, fractional exhaled nitric oxide, and asthma at school-age. *J Allergy Clin Immunol.* 2017;139(3):810-818.e816.

6. Joseph M, Elliott M, Zelicoff A, Qian Z, Trevathan E, Chang JJ. Racial disparity in the association between body mass index and self-reported asthma in children: a population-based study. *J asthma.* 2016;53(5):492-497.

7. Lai L, Zhang T, Zeng X, Tan W, Cai L, Chen Y. Association between Physician-Diagnosed Asthma and Weight Status among Chinese Children: The Roles of Lifestyle Factors. *Int J environ res public health.* 2020;17(5).

8. Sullivan PW, Ghushchyan V, Navaratnam P, et al. Exploring factors associated with health disparities in asthma and poorly controlled asthma among school-aged children in the U.S. *J Asthma.* 2020;57(3):271-285.

9. Vezir E, Civelek E, Dibek Misirlioglu E, et al. Effects of Obesity on Airway and Systemic Inflammation in Asthmatic Children. *Int archives allergy immunol.* 2021:1-11.

10. Zhang A, Li S, Zhang Y, Jiang F, Jin X, Ma J. Nocturnal enuresis in obese children: a nation-wide epidemiological study from China. *Sci reports.* 2019;9(1):8414.

11. Agnafors S, Norman Kjellstrom A, Torgerson J, Rusner M. Somatic comorbidity in children and adolescents with psychiatric disorders. *Eur Child Adolesc Psychiatry.* 2019;28(11):1517-1525.

12. Ahmadiafshar A, Ghoreishi A, Ardakani S, Khoshnevisasi P, Faghihhzadeh S, Nickmehr P. The high prevalence of depression among adolescents with asthma in Iran. *Psychosomatic Med.* 2016;78:113-117.

13. Arif AA, Korgaonkar P. The association of childhood asthma with mental health and developmental comorbidities in low-income families. *J Asthma.* 2016;53(3):277-281.

14. Barton CA, Dharmage SC, Lodge CJ, Abramson MJ, Erbas B, Lowe A. Asthma, atopy and serious psychological distress: prevalence and risk factors among young people in the Melbourne atopy cohort study. *J Asthma.* 2020;57(12):1323-1331.

15. Brew BK, Lundholm C, Gong T, Larsson H, Almqvist C. The familial aggregation of atopic diseases and depression or anxiety in children. *Clin Exp Allergy.* 2018;48(6):703-711.

16. Dogru H, Surer-Adanir A, Ozatalay E. Psychopathology, health-related quality-of-life and parental attitudes in pediatric asthma. *J Asthma.* 2019;56(11):1204-1211.

17. Dut R, Soyer O, Sahiner UM, et al. Psychological burden of asthma in adolescents and their parents. *J Asthma.* 2021:1-6.

18. Edvinsson Sollander S, Fabian H, Sarkadi A, et al. Asthma and allergies correlate with mental health problems in preschool children. *Acta Paediatr.* 2021;110(5):1601-1609.

19. Hammer-Helmich L, Linneberg A, Obel C, Thomsen SF, Tang Mollehave L, Glumer C. Mental health associations with eczema, asthma and hay fever in children: a cross-sectional survey. *BMJ Open.* 2016;6(10):e012637.

20. Kim CW, Jeong SC, Kim JY, et al. Associated factors for depression, suicidal ideation and suicide attempt among asthmatic adolescents with experience of electronic cigarette use. *Tob Induc Dis.* 2020;18:85.

21. Kyung Y, Han YJ, Lee JS, Lee JH, Jo SH, Kim SH. Evaluation of changing trend in depression, suicidal ideation, and suicide attempts among adolescents with asthma and identification of associated factors: 11-year national data analysis in 788,411 participants. *J Asthma.* 2021;58(7):921-931.

22. Kimura M, Ikeda A, Suzuki Y, Maruyama K, Wada H, Tanigawa T. The association between asthma and anxiety in elementary school students in Japan. *Pediatr Pulmonol.* 2020;55(10):2603-2609.

23. Laurens KR, Green MJ, Dean K, et al. Chronic Physical Health Conditions, Mental Health, and Sources of Support in a Longitudinal Australian Child Population Cohort. *J Pediatr Psychol.* 2019;44(9):1083-1096.

24. Mpairwe H, Mpango RS, Sembajjwe W, et al. Anxiety disorders and asthma among adolescents in Uganda: role of early-life exposures. *ERJ Open Res.* 2021;7(2).

25. Rajhans P, Sagar R, Patra BN, Bhargava R, Kabra SK. Psychiatric Morbidity and Behavioral Problems in Children and Adolescents with Bronchial Asthma. *Indian J Pediatr.* 2021.

26. van der Laan SEI, de Hoog MLA, Nijhof SL, et al. Mental Well-being and General Health in Adolescents with Asthma: The Prevention and Incidence of Asthma and Mite Allergy Birth Cohort Study. *J Pediatr.* 2021;233:198-205 e192.

27. Akmatov MK, Ermakova T, Batzing J. Psychiatric and Nonpsychiatric Comorbidities Among Children With ADHD: An Exploratory Analysis of Nationwide Claims Data in Germany. *J attention disorders.* 2021;25(6):874-884.

28. Chai PH, Chang S, Cawthorpe D. The Temporal Hyper-Morbidity of Asthma and Attention Deficit Disorder: Implications for Interpretation Based on Comparison of Prospective and Cross-Sectional Population Samples. *Psychiatry investigation.* 2021;18(2):166-171.

29. Chen M-H, Su T-P, Chen Y-S, et al. Comorbidity of Allergic and Autoimmune Diseases Among Patients With ADHD. *J attention disorders.* 2017;21(3):219-227.

30. Dai Y-X, Tai Y-H, Chang Y-T, Chen T-J, Chen M-H. Increased Risk of Atopic Diseases in the Siblings of Patients with Autism Spectrum Disorder: A Nationwide Population-Based Cohort Study. *J autism development disorders.* 2019;49(11):4626-4633.

31. Jameson ND, Sheppard BK, Lateef TM, Vande Voort JL, He J-P, Merikangas KR. Medical Comorbidity of Attention-Deficit/Hyperactivity Disorder in US Adolescents. *J child neuro.* 2016;31(11):1282-1289.

32. Jonsdottir U, Lang JE. How does autism spectrum disorder affect the risk and severity of childhood asthma? *Annals allergy, asthma & immunol.* 2017;118(5):570-576.

33. Strom MA, Fishbein AB, Paller AS, Silverberg JI. Association between atopic dermatitis and attention deficit hyperactivity disorder in U.S. children and adults. *Br J dermatol.* 2016;175(5):920-929.

34. van der Schans J, Pleiter JC, de Vries TW, et al. Association between medication prescription for atopic diseases and attention-deficit/hyperactivity disorder. *Annals allergy, asthma & immunol.* 2016;117(2):186-191.

35. Wang L-J, Yu Y-H, Fu M-L, et al. Attention deficit-hyperactivity disorder is associated with allergic symptoms and low levels of hemoglobin and serotonin. *Sci reports.* 2018;8(1):10229.

36. Weber RJ, Gadow KD. Relation of Psychiatric Symptoms with Epilepsy, Asthma, and Allergy in Youth with ASD vs. Psychiatry Referrals. *J abnormal child psychol.* 2017;45(6):1247-1257.

37. Xie L, Gelfand A, Delclos GL, Atem FD, Kohl HW, 3rd, Messiah SE. Estimated Prevalence of Asthma in US Children With Developmental Disabilities. *JAMA network open.* 2020;3(6):e207728.

38. Yang C-F, Yang C-C, Wang IJ. Association between allergic diseases, allergic sensitization and attention-deficit/hyperactivity disorder in children: A large-scale, population-based study. *J Chinese Med Assoc.* 2018;81(3):277-283.

39. Andersen IG, Holm J-C, Homoe P. Obstructive sleep apnea in children and adolescents with and without obesity. *Eur archives oto-rhino-laryngol.* 2019;276(3):871-878.

40. Dooley AA, Jackson JH, Gatti ML, et al. Pediatric sleep questionnaire predicts more severe sleep apnea in children with uncontrolled asthma. *J Asthma.* 2020:1-8.

41. Estanislau NRdA, Jordao EAdOC, Abreu GdA, et al. Association between asthma and sleep hours in Brazilian adolescents: ERICA. *Jornal de pediatria.* 2020.

42. Garden M, O'Callaghan M, Suresh S, Mamum AA, Najman JM. Asthma and sleep disturbance in adolescents and young adults: A cohort study. *J paeds child health.* 2016;52(11):1019-1025.

43. Guo Y, Pan Z, Gao F, et al. Characteristics and risk factors of children with sleep-disordered breathing in Wuxi, China. *BMC pediatrics.* 2020;20(1):310.

44. Kilaikode S, Weiss M, Megalaa R, Perez G, Nino G. Asthma is associated with increased probability of needing CPAP in children with severe obstructive sleep apnea. *Pediatric pulmonol.* 2019;54(3):342-347.

45. Martin SR, Boergers J, Kopel SJ, et al. Sleep Hygiene and Sleep Outcomes in a Sample of Urban Children With and Without Asthma. *J pediatr Psychol.* 2017;42(8):825-836.

46. Meltzer LJ, Pugliese CE. Sleep in young children with asthma and their parents. *J Child health care.* 2017;21(3):301-311.

47. Narayanan A, Yogesh A, Mitchell RB, Johnson RF. Asthma and obesity as predictors of severe obstructive sleep apnea in an adolescent pediatric population. *The Laryngoscope.* 2020;130(3):812-817.

48. Nguyen-Hoang Y, Nguyen-Thi-Dieu T, Duong-Quy S. Study of the clinical and functional characteristics of asthmatic children with obstructive sleep apnea. *J asthma allergy.* 2017;10:285-292.

49. Perikleous E, Steiropoulos P, Nena E, et al. Association of Asthma and Allergic Rhinitis With Sleep-Disordered Breathing in Childhood. *Frontiers in pediatrics.* 2018;6:250.

50. Reynolds KC, Boergers J, Kopel SJ, Koinis-Mitchell D. Featured Article: Multiple Comorbid Conditions, Sleep Quality and Duration, and Academic Performance in Urban Children With Asthma. *J pediatr psychol.* 2018;43(9):943-954.

51. Tamanyan K, Walter LM, Davey MJ, Nixon GM, Horne RS, Biggs SN. Risk factors for obstructive sleep apnoea in Australian children. *J Paeds Child Health.* 2016;52(5):512-517.

52. Zaffanello M, Gasperi E, Tenero L, et al. Sleep-Disordered Breathing in Children with Recurrent Wheeze/Asthma: A Single Centre Study. *Children.* 2017;4(11).

53. Zandieh SO, Cespedes A, Ciarleglio A, Bourgeois W, Rapoport DM, Bruzzese J-M. Asthma and subjective sleep disordered breathing in a large cohort of urban adolescents. *J Asthma.* 2017;54(1):62-68.

54. Bourne T, Waltz M, Casper TC, et al. Evaluating the association of allergies with multiple sclerosis susceptibility risk and disease activity in a pediatric population. *J Neurological Sci.* 2017;375:371-375.

55. Ghersin I, Khateeb N, Katz LH, Daher S, Shamir R, Assa A. Comorbidities in adolescents with inflammatory bowel disease: findings from a population-based cohort study. *Pediatric research.* 2020;87(7):1256-1262.

56. Kim SY, Min C, Oh DJ, Choi HG. Increased risk of psoriasis in children and elderly patients with asthma: a longitudinal follow-up study using a national sample cohort. *Int forum allergy & rhinology.* 2019;9(11):1304-1310.

57. Kuenzig ME, Barnabe C, Seow CH, et al. Asthma Is Associated With Subsequent Development of Inflammatory Bowel Disease: A Population-based Case-Control Study. *Clinical gastroentero hepatol.* 2017;15(9):1405-1412.e1403.

58. Lin C-H, Lin C-L, Shen T-C, Wei C-C. Epidemiology and risk of juvenile idiopathic arthritis among children with allergic diseases: a nationwide population-based study. *Pediatric rheumatol.* 2016;14(1):15.

59. Metsala J, Lundqvist A, Virta LJ, et al. Use of Antiasthmatic Drugs and the Risk of Type 1 Diabetes in Children: A Nationwide Case-Cohort Study. *Am J Epidemiol.* 2020;189(8):779-787.

60. Metsala J, Lundqvist A, Virta LJ, et al. The association between asthma and type 1 diabetes: a paediatric case-cohort study in Finland, years 1981-2009. *Int J Epidemiol.* 2018;47(2):409-416.

61. Patel B, Wi C-I, Hasassri ME, et al. Heterogeneity of asthma and the risk of celiac disease in children. *Allergy asthma proc.* 2018;39(1):51-58.

62. Smew AI, Lundholm C, Savendahl L, Lichtenstein P, Almqvist C. Familial Coaggregation of Asthma and Type 1 Diabetes in Children. *JAMA network open.* 2020;3(3):e200834.

63. Wasielewska Z, Dolinska A, Wilczynska D, Szaflarska-Poplawska A, Krogulska A. Prevalence of allergic diseases in children with inflammatory bowel disease. *Postepy dermatologii i alergologii.* 2019;36(3):282-290.
